# The Phytochemical Composition of *Melia volkensii* and Its Potential for Insect Pest Management

**DOI:** 10.3390/plants9020143

**Published:** 2020-01-22

**Authors:** Victor Jaoko, Clauvis Nji Tizi Taning, Simon Backx, Jackson Mulatya, Jan Van den Abeele, Titus Magomere, Florence Olubayo, Sven Mangelinckx, Stefaan P.O. Werbrouck, Guy Smagghe

**Affiliations:** 1 Department of Plants and Crops, Ghent University, Coupure Links 653, B-9000 Ghent, Belgium; tiziclauvis.taningnji@ugent.be (C.N.T.T.); guy.smagghe@ugent.be (G.S.); 2 SynBioC, Department of Green Chemistry and Technology, Ghent University, Coupure Links 653, B-9000 Ghent, Belgium; simon.backx@ugent.be; 3 Kenya Forestry Research Institute, P.O Box 20412-00200 Nairobi, Kenya; jmulatya@kefri.org; 4 Better Globe Forestry, P.O Box 823-00606 Nairobi, Kenya; jan@betterglobeforestry.com; 5 Department of Plant Science and Crop Protection, University of Nairobi, P.O Box 30197-0010 Nairobi, Kenya; magomere.titus@ku.ac.ke (T.M.); olubayo@uonbi.ac.ke (F.O.)

**Keywords:** Meliaceae, *Melia volkensii*, botanical pesticide, limonoid, insect pest, antifeedant, growth inhibitor

## Abstract

Due to potential health and environmental risks of synthetic pesticides, coupled with their non-selectivity and pest resistance, there has been increasing demand for safer and biodegradable alternatives for insect pest management. Botanical pesticides have emerged as a promising alternative due to their non-persistence, high selectivity, and low mammalian toxicity. Six Meliaceae plant species, *Azadirachta indica*, *Azadirachta excelsa*, *Azadirachta siamens*, *Melia azedarach*, *Melia toosendan*, and *Melia volkensii*, have been subject to botanical pesticide evaluation. This review focuses on *Melia volkensii*, which has not been intensively studied. *M. volkensii*, a dryland tree species native to East Africa, has shown activity towards a broad range of insect orders, including dipterans, lepidopterans and coleopterans. Its extracts have been reported to have growth inhibiting and antifeedant properties against *Schistocerca gregaria*, *Trichoplusia ni*, *Pseudaletia unipuncta*, *Epilachna varivestis*, *Nezara viridula*, several *Spodoptera* species and other insect pests. Mortality in mosquitoes has also been reported. Several limonoids with a wide range of biological activities have been isolated from the plant, including volkensin, salannin, toosendanin, trichilin-class limonoids, volkendousin, kulactone among others. This paper presents a concise review of published information on the phytochemical composition and potential of *M. volkensii* for application in insect pest management.

## 1. Introduction

The continuous and indiscriminate use of synthetic pesticides in crop protection has led to an increase in pest resistance, health and environmental concerns [1]. This has led to a renewed interest in natural products as alternative sources for insect pest control [1]. One of the most promising options is the use of secondary metabolites produced by plants, many of which are toxic to a wide spectrum of insect pests [2]. Plant extracts can offer a solution to insect pest control because they are environmentally friendly, easily biodegradable, and are target-specific [3]. 

The *Meliaceae* plant family has been reported to produce a wide range of compounds, including flavonoids, chromones, coumarins, benzofurans, mono-, sesqui-, di-, and triterpenoids, but tetranortriterpenoids with a β-substituted furanyl ring at C17α are the best known for the production of limonoids [4]. Limonoids are known for a range of biological activities, including insect antifeedant and growth-regulating properties and antibacterial properties [4]. Alkaloids are rarely isolated from Meliaceae [4]. Reviews on the Meliaceae plant family have been reported in the literature. The use of Meliaceae plant extracts as potential mosquitocides have been reviewed, and *Azadirachta indica* A. Juss (Indian neem tree) is reported as a potential plant for the control of vector mosquitoes [5]. Reviews on the chemical constituents of the genus Melia reported the isolation of terpenoids, steroids, alkaloids, flavonoids, anthraquinones with a wide range of biological activities including antiviral, pesticidal, inhibition of iNOS, antitumor, antibacterial and antifungal activities [6,7]. A phytopharmacological review of *Melia azedarach* Linnaeus (chinaberry) has been reported outlining its use in folk medicine having antifertility, antiviral, cytotoxic, antibacterial, immunomodulatory, repellent, antifeedant, antilithic and anthelmintic activity from various parts of the plant [8,9]. A review on *A. indica* has reported its use in agriculture for application as manure, fertilizer, soil conditioner, fumigant, and as botanical pesticide [10]. *Melia volkensii* (Gurke) has also been identified as one of the pesticidal plants in Africa [11]. Another review has explored the phytochemical and antimicrobial activities of the Meliaceae family [12]. Detailed information on commercially available neem products developed for agricultural pest control has also been reviewed [13].

Several plant species of the Meliaceae have shown promising bioactivity against a variety of insects [3]. Their insect growth regulatory and antifeedant properties against many insect pests have made them emerge as a potent source of insect control products [14]. Six species have been subjected to botanical pesticide evaluation; these include *A. indica* (Indian neem tree), *Azadirachta excelsa* Jack (Philippine neem tree), *Azadirachta siamens* Valeton (Siamese neem tree), *M. azedarach* (chinaberry), *Melia toosendan* Siebold and Zucc., and *M. volkensii* [13]. However, research has concentrated mostly on *A. indica* (neem tree) and *M. azedarach* (chinaberry) [15]. Azadirachtin, a commercial biopesticide, and other limonoids isolated from *A. indica*, have been effective growth regulators and feeding deterrents for a wide range of insect species [16]. Azadirachtin targets the corpus cardiacum in insects, which in turn affects neuroendocrine activity and turnover of neurosecretion [17]. Extracts from *M. azedarach* have also shown antifeedant activity against the juvenile and adult *Xanthogaleruca luteola* Muller (elm leaf beetles) and mortality against its larvae [16]. Fruit extracts from *M. azedarach* are also effective against *Napomyza lateralis* Fallen (agromyzid leafminers) and *Trialeurodes vaporariorum* Westwood (whiteflies) [16]. Toosendanin, a limonoid constituent of *M. azedarach* which has been commercialized in China, is an effective growth inhibitor against *Ostrinia nubilalis* Hübner (European corn borer), effective repellent against *Pieris brassicae* Linnaeus (cabbage moth) and an oviposition deterrent against *Trichoplusia ni* Hübner (cabbage looper) [16]. Toosendanin is reported to be mainly active against lepidopteran pests and is less active than azadirachtin [18].

**Figure 1:**
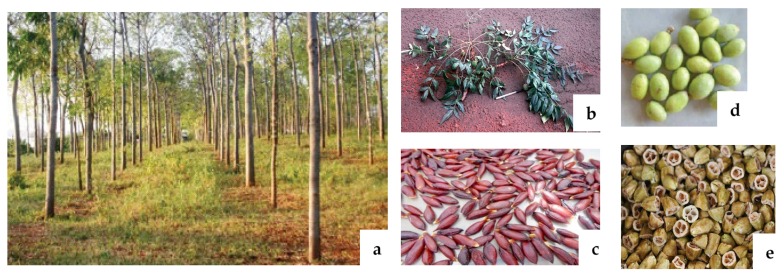
*Melia volkensii* and its various parts: (**a**) 10-year old *M. volkensii* plantation, (**b**) leaves, (**c**) seeds, (**d**) fruits and (**e**) nuts [23].

*M. volkensii*, a dryland tree species native to East Africa has, however, not been intensively studied [16]. It is a tall tree (15–25 m), shown in Figure 1, which grows in semi-arid areas of Kenya, Tanzania, Ethiopia, and Somalia at altitudes of between 350 to 1700 m above sea level [19]. The tree, like other meliaceous plants, is fast growing and produces fruits after 4–5 years [19]. It remains green for most of the year and is prized by farmers for its termite-resistant timber. It is intercropped with food crops, used for shade, firewood, and livestock fodder [19]. Several chemical compounds occur only in *M. volkensii*. These include: 1-O-cinnamoyltrichilin, meliavolkinin, 1,3-diacetylvilasinin, meliavolkin, volkensin, volkensinin, 12β- and 6β-hydroxykulactone, meliavolkenin, meliavolin, meliavolen, melianinone, meliavolkensin A and B, melianin C, (E)- and (Z)-volkendousin, meliavosin, 2-9-epoxymeliavosin [6]. *M. volkensii* seed kernel extracts have more insect growth inhibitory and acute lethal toxicity than azadirachtin-containing fractions from neem seed kernel extracts [20]. It has been reported that when *M. volkensii* dried fruit powder and residual fruit cake obtained after extraction with ethanol are used as goat feed, their growth and performance are not negatively affected, indicating that both fruit powder and its cake could be used as safe ruminant feed supplement [21]. Its use as a fodder crop underscores its safety in mammals [20], and traditionally, it is used for the treatment of diarrhea, pain, skin rashes, and eczema [22]. Aqueous extracts of *M. volkensii* have also traditionally been used to control ticks and fleas in goats [19]. *M. volkensii* offers a key indigenous tree species that can be used to mitigate against desertification in arid and semi-arid lands [23], while also offering a high economic potential for the rural community in these regions [24]. This paper presents a concise review of published information on the phytochemical composition and potential application of *M. volkensii* in insect pest management.

## 2. Biological Activity of *Melia volkensii* Extracts Against Insects

Crude fruit extracts from *M. volkensii* have been reported to pose activity towards a broad range of insect orders including Diptera, Lepidoptera, Coleoptera among others [19] as shown in Table A1 (Appendix A). The methanolic fruit extracts were first reported to have antifeedant effects against *Schistocerca gregaria* Forsk. (desert locusts) [25]. Repellency effect, decreased mobility, retarded development and reduced fecundity were observed against *S. gregaria* when seed extract was applied to their preferred host plants mainly *Schouwia thebaica* Webb, *Fagonia olivieri* DC (fagonbush plant) and *Hyoscyamus muticus* Linnaeus (Egyptian henbane) in a field trial experiment [26]. Although the mode of action of the extracts is still unknown, it is postulated that the active compounds in *M. volkensii* extracts could affect hormone levels in *S. gregaria* larvae [27]. In fifth-instar nymphs of *S. gregaria*, 80% mortality was recorded 48 hours after injection with crude ethanolic and methanolic extracts at a concentration of 30 μg/g of the insect [19]. When sprayed on third- to fifth-instar *S. gregaria*, *M. volkensii* and neem oil have been reported to cause mortality of up to 91% and 92%, respectively, after 14 days in a comparative study [26]. In contrast to synthetic pesticides, these botanicals do not have a knock-down effect, but their slow response is similar to inhibitors of chitin synthesis [26]. 

Antifeedant and larval growth inhibitory effects of fruit extracts have been observed in *Trichoplusia ni* Hübner (cabbage looper) and *Pseudaletia unipuncta* Haworth (true armyworm) [25,28]. Crude seed extracts are also an effective growth inhibitor against *T. ni* (dietary EC_50_ = 7.6 ppm) and feeding deterrent (DC_50_ = 0.9 μg/cm^2^) [29]. Prolonged exposure to *M. volkensii* extracts has been observed to lead to a decrease in antifeedant response when tested against *T. ni* implying that the insect could develop tolerance to the extracts [30]. However, when tested against *Plutella xylostella* Linnaeus (diamondback moth) and *P. unipuncta*, there was no significant decrease in feeding deterrent response to the extracts following continuous exposure [31]. It has been postulated that triterpenoids from seed kernels of *M. volkensii* are responsible for the insecticidal activity in *T. ni* [11]. Comparative efficacy has been observed with *M. volkensii* extracts, other Meliaceae plant extracts (*A. indica*, *A. excelsa*, *M. azedarach*, and *Trichilia americana* Sessé & Mocino) and commercial botanical insecticides (ryania, pyrethrum, rotenone and essential oils of rosemary and clove leaf) when tested against *T. ni* and *P. unipuncta* [32]. 

*M. volkensii* fruit extracts when tested at concentrations ranging from 1 to 50 μg/μL showed feeding deterrence, growth disruption and mortality against *Nezara viridula* Linnaeus (stink bug), a polyphagous pest which attacks a variety of crops, including nuts, corn, cotton, grains and tomatoes [16]. The disruption of the molting process led to eventual mortality in *N. viridula* [16]. Furthermore, deformities and malfunctions like shortened or missing antennae, legs failing to detach from the exuvium, absent or shortened hemelytra, notching, and lack of symmetry have been observed in *N. viridula* when exposed to fruit extracts, with 10 μg/μL causing malformation in up to 85.70% of surviving adults [16]. A delay of the imaginal molt was observed in immature *Coranus arenaceus* Walker even though there were no deformities in resultant adults after topical application of the *M. volkensii* extracts at 1, 5, and 10 μg/μL [16]. 

When applied to cabbage leaf disks in a choice bioassay, *M. volkensii* fruit extract showed potent antifeedant properties against *Epilachna varivestis* Mulsant (Mexican bean beetle) [16]. Growth inhibition has also been observed in *P. unipuncta* (dietary EC_50_ = 12.5 ppm) with refined seed extracts to the leaf discs in a choice bioassay [29]. The seed extracts also showed feeding deterrent effects on third-instar larvae of *P. unipuncta* and *P. xylostella*, and adults of *E. varivestis* (DC_50_ = 10.5, 20.7 and 2.3 μg/cm^2^, respectively) [29]. In fact, *M. volkensii* seed extracts have been recorded to have stronger antifeedant activity compared to pure allelochemicals: digitoxin, cymarin, xanthotoxin, toosendanin, thymol and *trans*-anethole against *P. unipuncta*, *P. xylostella* and *E. varivestis* [29]. When applied to *Spodoptera litura* Fabricius, neem, rotenone, *M. volkensii* extract, toosendanin, *Annona squamosal* L. extract and pyrethrum at 1% concentration recorded larval growth (% relative to control) of 4.1, 97.5, 26.2, 48.3, 61.4, and 56.6%, respectively after 96 h in a comparative study [1].

Dried *M. volkensii* fruit extracts have shown growth-inhibiting activity against *Aedes aegypti* Linnaeus (yellow fever mosquito) larvae at 2 μg/mL in water, whilst recording high mortality during the molting and melanization process with LC_50_ of 50 μg/mL in 48 h [13]. At a high dose (100 μg/mL), the extracts caused acute toxicity, while at a low dose, the lethal effect took a long time, indicating the presence of compounds with an acute toxic effect at a high concentration and a growth-inhibiting effect at a low concentration [20]. Growth inhibiting and disrupting effects in *A. aegypti* could be a result of synergistic effects of a plethora of limonoid compounds or a single active compound exerting these effects [20]. 

A column chromatography-purified fraction of *M. volkensii* fruit kernel extract showed growth-inhibiting activity against *Anopheles arabiensis* Giles with an LC_50_ of 5.4 μg/mL in 48 h [13]. Mortality (LC_50_ of 34.72 μg/mL in 48 h) and oviposition deterrence was observed in second-instar larvae of *Culex quinquefasciatus* Say (Southern house mosquito) when treated with refined methanolic fruit extracts [33]. The granular formulation of *M. volkensii* fruit acetone extract showed S- and U-shaped postures and frequent stretching in *C. quinquefasciatus*; such postures and stretching are a characteristic of mosquito larvae reared in *M. volkensii* fruit extract [34]. The test granules also caused 86% mortality in third- and fourth-instar larvae of *C. quinquefasciatus* within 36 h [34]. Acetone extracts from *M. volkensii* seeds have recorded growth inhibitory effects and equal toxicity (LD_50_ of 30 μg/mL) for larvae and pupae of *C. pipiens* f. molestus Forskål (London underground mosquito) [17]. *M. azedarach* seed extracts recorded lower toxicity (LD_50_ of 40 μg/mL) while pure azadirachtin A recorded higher toxicity (LD_50_ of 1–5 μg/mL) against *C. pipiens* when compared with *M. volkensii* extracts [17]. The water solubility of the acetone seed extract from *M. volkensii* may indicate the presence of saponins as toxic principles thus making it an interesting candidate for application against aquatic insects such as mosquitoes and other vectors of diseases [17].

## 3. Phytochemistry and Insect Bioactivity of *Melia volkensii*

**Figure 2:**
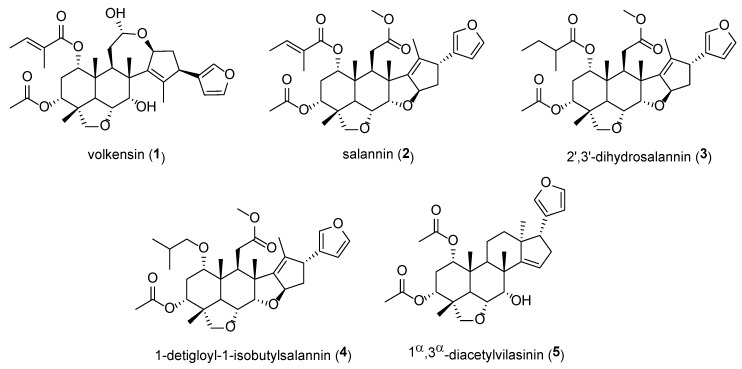
Chemical structures of compounds isolated from *Melia volkensii* with antifeedant and growth-inhibition activity against insects.

Insect antifeedants have been found in major classes of secondary metabolites—alkaloids, phenolics, and terpenoids [35]. However, it is in the terpenoids that the greatest number and diversity of antifeedants, and the most potent, have been found. Most well-documented antifeedants are triterpenoids [35]. Effective insect antifeedants have been isolated from various parts of *M. volkensii*, as shown in Figure 2 and Table A2 (Appendix B), although azadirachtin, the major ingredient in neem seeds, does not occur in *M. volkensii*. This indicates that insect control bioactivity is, therefore, based on other compounds than azadirachtin [25]. It is postulated that the major active compound in *M. volkensii* fruit is more lipophilic than azadirachtin [20]. Botanical antifeedants are easily degraded after application thereby causing little environmental impact [36].

The insect antifeedants volkensin (**1**) and salannin (**2**) have been isolated from seed extracts of *M. volkensii* [37]. Additionally, volkensin (**1**) and salannin (**2**) were isolated from the whole fruits of *M. volkensii* [37]. Volkensin (**1**) has shown antifeedant activity against *Spodoptera frugiperda* Smith (fall armyworms) larvae with an ED_50_ of 3.5 μg/cm^2^ [19]. Salannin (**2**) has also shown antifeedant activity against insect pests such as *Acalymma vittata* Fabricius (striped cucumber beetle), *Musca domestica* Linnaeus (housefly), *Epilachna varivestis* Mulsant (Mexican bean beetle), *Heliothis virescens* Fabricius (tobacco budworm), *S. frugiperda* and *Spodoptera littoralis* Boisduval (cotton leafworm) [38]. Salannin (**2**) has also been reported to cause feeding suppression against larvae of *Earias insulana* Boisduval (Egyptian stemborer), weight reduction (59%–89%) in *Cnaphalocrocis medinalis* Guenee (rice leafroller) and reduction in activities of acid phosphatases (ACP), alkaline phosphatases (ALP) and adenosine triphosphatases (ATPase), implying that gut enzyme activities were affected. 2’,3’-Dihydrosalannin (**3**), 1-detigloyl-1-isobutylsalannin (**4**) and 1α,3α-diacetylvilasinin (**5**) have also been isolated from the plant [7].

**Figure 3:**
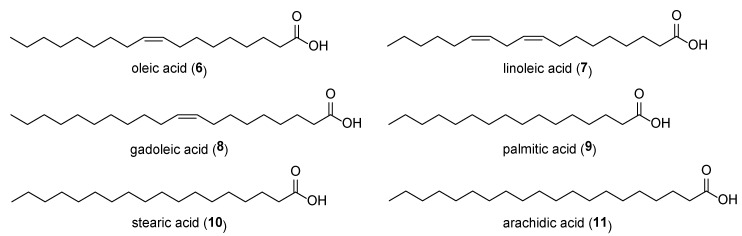
Chemical structures of saturated and unsaturated fatty acids isolated from *Melia volkensii*.

*M. volkensii* seed extracts, extracted in cold water, have been reported to contain unsaturated fatty acids (oleic acid (**6**), linoleic acid (**7**) and gadoleic acid (**8**)) and saturated fatty acids (palmitic acid (**9**), stearic acid (**10**) and arachidic acid (**11**)) as shown in Figure 3 [39]. Fatty acids with at least 18 carbon atoms have been found to synergistically enhance insecticidal activity of insecticides [40]. Oleic acid (**6**), linoleic acid (**7**), linolenic acid, and ricinoleic acid have enhanced insecticidal activity of organophosphates and carbamates when applied against sucking insects and defoliating insects [40]. 

**Figure 4:**
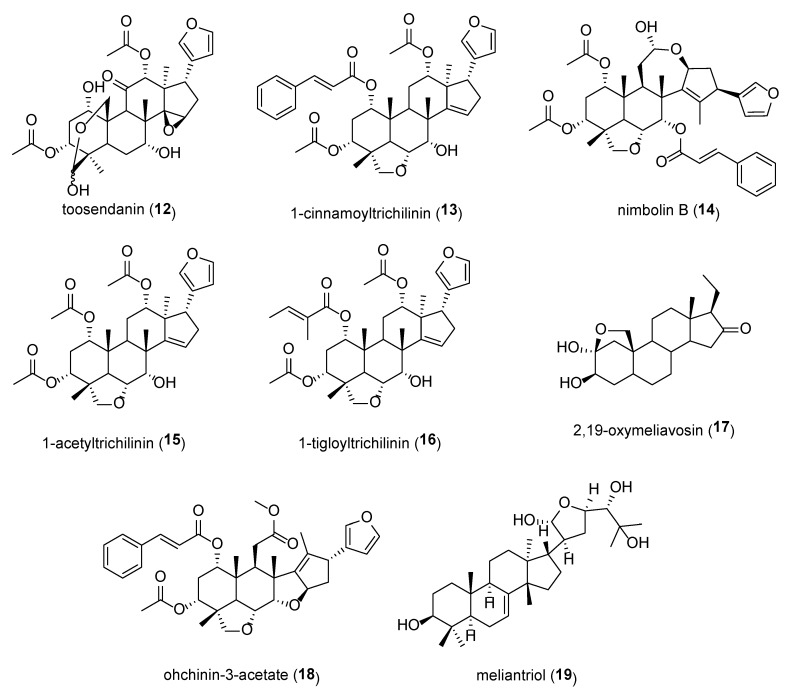
Further chemical structures of compounds isolated from *Melia volkensii* with antifeedant and growth-inhibition activity against insects.

Other chemical compounds that have been isolated from various parts of *M. volkensii* are shown in Figure 4. Toosendanin (**12**), which has been isolated from the root bark of *M. volkensii* [22], has been reported to be an effective growth inhibitor against *O. nubilalis*, an effective repellent against *P. brassicae* and an oviposition deterrent against *T. ni* [16]. 1-Cinnamoyltrichilinin (**13**) has shown antifeedant activity towards *S. littoralis* having minimum antifeedant concentration (MAC) value of 1000 mg/L and a significant antibacterial activity against *Porphyromonas gingivalis* ATCC 33277 with minimum inhibitory concentration (MIC) value of 15.6 μg/mL [7]. Nimbolin B (**14**) has been reported to have antifeedant activity against several *Spodoptera* species (*S. exigua*, *S. eridania* and *S. littoralis*) [7]. There was a clear-cut structure-activity relationship when trichilin-class limonoids (1-cinnamoyltrichilinin **13**, 1-acetyltrichilinin **15**, 1-tigloyltrichilinin **16**) were tested against *Spodoptera eridania* Stoll (Southern armyworm) where the 12α-OH function was the most potent, followed by 12β-OH, 12-desoxy, and 12α-acetoxy groups in order of decreasing potency [7]. The 12-OH functionality could be necessary for maximum bioactivity in trichilin-class limonoids (**13**, **15**, **16**) [7]. 2,19-oxymeliavosin **17**, which has weak activity with marginally significant selectivity for breast cancer cell line (MCF-7), has also been isolated from the root bark of *M. volkensii* [41]. Ohchinin-3-acetate (**18**), isolated from methanolic extract of *M. volkensii* fruits [42], and meliantriol (**19**), both insect antifeedants have also been reported [15]. Meliantriol has exhibited moderate cytotoxicity against human epidermoid carcinoma of the nasopharynx (KB), multidrug-resistant (KB-C2), and breast cancer cell line (MCF-7) [43]. 

## 4. Further Phytochemical Composition and Biological Activity of *Melia volkensii*

Other compounds have also been isolated from *M. volkensii* with different biological activities. These include volkensinin, as isolated from ethanolic extracts of *M. volkensii* root bark [44], which showed weak bioactivity in the brine shrimp lethality test BST (LC_50_ = 57 μg/mL) and weak cytotoxicity against six human tumor cell lines with ED_50_ values of 27.90, 28.35, 33.56, 29.55, 8.43, and 28.51 μg/mL in A-498 (human kidney carcinoma), PC-3 (prostate adenocarcinoma), PACA-2 (pancreatic carcinoma), A-549 (human lung carcinoma), MCF-7 (human breast carcinoma), and HT-29 (human colon adenocarcinoma), respectively [44]. Toosendanin has activity against *Escherichia coli* Migula and *Aspergillus niger* Tiegh. with respective minimum inhibitory concentration (MIC) values of 12.5 and 6.25 μg/mL [22]. Melianin B, isolated from the root bark of *M. volkensii*, showed cytotoxicity against six human solid tumor cell lines: A-549, MCF-7, HT-29, A-498, PACA-2, and PC-3 [45]. Bioactivity-guided fractionation of *M. volkensii* root bark led to the isolation of meliavolkenin which showed moderate cytotoxicity against three human tumor cell lines with a respective ED_50_ value of 10.33 μg/mL, 4.30 μg/mL, and 0.67 μg/mL in A-549, MCF-7, and HT-29 cells [46]. The bioactive apotirucallane triterpenes meliavolkensin A and meliavolkensin B, both isolated from the root bark of *M. volkensii* [47], have shown cytotoxicity against human colon tumor cell lines H-29 (human colon adeno-carcinoma) with ED_50_ values of 0.49 μg/mL and 0.25 μg/mL, respectively [47]. (*E*)-volkendousin, isolated from *M. volkensii* root bark, also showed activity against six human tumor cell lines (A-549, MCF-7, HT-29, A-498, PACA-2 and PC-3) [48]. Meliavolin, marginally cytotoxic against human tumor cell lines with an ED_50_ of 11.25 μg/mL, 0.57 μg/mL and 6.65 μg/mL in A-549, MCF-7 and HT-29 cells, respectively [49], has been isolated from *M. volkensii* root bark following activity-directed fractionation with brine shrimp test [49]. Kulactone was isolated from root bark of *M. volkensii* and exhibited significant activity against *E. coli* and *A. niger* with a respective minimum inhibitory concentration (MIC) value of 12.5 and 6.25 μg/mL [22]. Bioactivity-guided antimycobacterial investigations against *Mycobacterium tuberculosis* Zopf resulted in the isolation of 12β-hydroxykulactone, 6β-hydroxykulactone and kulonate from *M. volkensii* seeds with MIC values of 16 μg/mL, 4 μg/mL, and 16 μg/mL, respectively [50]. Meliavolkin has shown anticancer activity against three human tumor cell lines: A-549 (ED_50_ = 0.57 μg/mL), MCF-7 (ED_50_ = 0.26 μg/mL), and HT-29 (ED_50_ = 0.12 μg/mL) [7]. Other limonoids isolated from *M. volkensii* include 3-episapelin, meliavolen, melianinone [4], and nimbolin B [51] and all have shown selectivity for the colon cell line HT-29 [51]. Other compounds, which have been isolated from *M. volkensii* include scopoletin [22], melianin C and meliavolkinin [7], methyl kulonate and 2,19-epoxymeliavosin [6], nimbolidins C-E [12]. However, their activity against insects has not been reported in literature. 

## 5. Conclusions

Extracts and pure compounds isolated from *M. volkensii* have proved to be effective insect antifeedants and growth inhibitors. Extensive research has been done on mosquito control using *M. volkensii*; however, more research needs to be done on insect pests of agricultural importance. *M. volkensii* has no reported adverse effect on the environment or mammals, making it a potential botanical pesticide for the biosafe application in integrated pest management. The availability of renewable resources from the tree, such as fruits, stem bark, and leaves makes this plant a potential candidate for insect control with minimal interference on the plant. In this regard, *M. volkensii* could be further exploited as a source of natural insecticide. 

## Appendix A

**Table A1:** *Melia volkensii* as a botanical pesticide for insect pest control.

Target Insect *	Order	Biological Activity	Plant Part Used	Reference
Desert locust, *Schistocerca gregaria*	Orthoptera	Antifeedant, repellency, growth inhibition, mortality	Fruit	[19,25,26]
Cabbage looper, *Trichoplusia ni*	Lepidoptera	Antifeedant, growth inhibition, mortality	Fruit, seed	[25,28,29,30]
True armyworm, *Pseudaletia unipuncta*	Lepidoptera	Antifeedant, growth inhibition	Fruit, seed	[11,25,28,29,31]
Diamondback moth, *Plutella xylostella*	Lepidoptera	Antifeedant	Fruits	[29,31]
Stink bug, *Nezara viridula*	Hemiptera	Antifeedant, growth disruption, mortality	Fruit	[16]
*Coranus arenaceus*	Hemiptera	Growth inhibition	Fruit	[16]
Mexican bean beetle, *Epilachna varivestis*	Coleoptera	Antifeedant, growth inhibition	Seed	[16,29]
Yellow fever mosquito, *Aedes aegypti*	Diptera	Growth inhibition, mortality	Fruit	[13,20]
*Anopheles arabiensis*	Diptera	Growth inhibition	Fruit kernel	[13]
Southern house mosquito, *Culex quinquefasciatus*	Diptera	Oviposition deterrence, mortality	Fruit	[13,33,34]
London underground mosquito, *Culex pipiens molestus*	Diptera	Growth inhibition, mortality	Seed	[17]

* Non exhaustive list of potential target insect pests.

## Appendix B

**Table A2:** Phytochemical investigation of *Melia volkensii*.

Compound *	Plant Part Isolated From	Biological Activity	Reference
Volkensin	Seed, fruit	Antifeedant against fall armyworms, *Spodoptera frugiperda*	[19,37]
Salannin	Seed, fruit	Antifeedant and weight reduction against *Acalymma vittata, Musca domestica, Epilachna varivestis, Heliothis virescens, Spodoptera frugiperda, Earias insulana, Cnaphalocrocis medinalis* and *Spodoptera littoralis*	[7,37,38]
Toosendanin	Root bark	Growth inhibitor and oviposition deterrent against *Ostrinia nubilalis, Pieris brassicae, Trichoplusia ni*	[16,22]
Meliantriol	Not reported	Antifeedant	[15]
Unsaturated fatty acids (oleic acid, linoleic acid and gadoleic acid); saturated fatty acids (palmitic acid, stearic acid and arachidic acid)	Seed	Synergistic enhancement of insecticidal activity	[39,40]
1-cinnamoyltrichilinin	Not reported	Antifeedant against *Spodoptera littoralis*	[7]
1-tigloyltrichilinin	Not reported	Antifeedant against *Spodoptera eridania*	[7]
1-acetyltrichilinin	Not reported	Antifeedant against *Spodoptera eridania*	[7]
Nimbolin B	Not reported	Antifeedant against *Spodoptera* species. (*exigua, eridania* and *littoralis*)	[7,51]
Ohchinin-3-acetate	Fruit	Antifeedant	[42]

* Non exhaustive list of compounds present in *M. volkensii*.
